# Supplementary material for: Saponin Fraction CIL1 from Lysimachia ciliata L. Enhances the Effect of a Targeted Toxin on Cancer Cells
Source: Pharmaceutics. 2023 Apr 28;15(5):1350. doi: 10.3390/pharmaceutics15051350 (PMC10221382; doi:10.3390/pharmaceutics15051350)
Supplement: Supplementary file 1 [file pharmaceutics-15-01350-s001.zip › pharmaceutics-2348173-supplementary.pdf]

pharmaceutics

Supplementary Material: Saponin fraction CIL1 from *Lysimachia ciliata* L. Enhances the Effect of a Targeted Toxin on Cancer Cells

Paulina Koczurkiewicz-Adamczyk, Karolina Grabowska, Elżbieta Karnas, Kamil Piska, Dawid Wnuk, Katarzyna Klaś, Agnieszka Galanty, Katarzyna Wójcik-Pszczoła, Marta Michalik, Elżbieta Pękala, Hendrik Fuchs and Irma Podolak

UPLC/MS/MS analysis

The UPLC-MS/MS system consisted of a Waters ACQUITY® UPLC® (Waters Corporation, Milford, MA, USA) coupled to a Waters TQD mass spectrometer (electrospray ionization mode ESI negative-tandem quadrupole). Chromatographic separations were carried out using an Acquity UPLC BEH (bridged ethyl hybrid) C_{18} column (2.1 × 100 mm) with a 1.7 μm particle size, equipped with an Acquity UPLC BEH C18 VanGuard pre-column (2.1 × 5 mm) with a 1.7 μm particle size. The column was maintained at 40°C and eluted under gradient conditions: from 95% to 0% of eluent A over 10 min at a flow rate of 0.3 mL min^{-1}. Eluent A: water/formic acid (0.1%, v/v) and eluent B: acetonitrile/formic acid (0.1%, v/v).

The MS detection settings of the Waters TQD mass spectrometer were as follows: source temperature, 150 °C; desolvation temperature, 350°C; desolvation gas flow rate, 600 L h^{-1}; cone gas flow, 100 L h^{-1}; capillary potential, 3.00 kV; and cone potential, 30 V. Nitrogen was used as both a nebulizing and drying gas. The data were obtained in a scan mode ranging from 50 to 2000 m/z in 1.0 s intervals; 8 scans were summed to obtain the final spectrum.

Collision activated dissociations (CAD) analyses were carried out with energies of 60 and 80 eV. Consequently, the ion spectra were obtained by scanning in the 100 to 1200 m/z range. The data acquisition software was MassLynx V 4.1 (Waters).

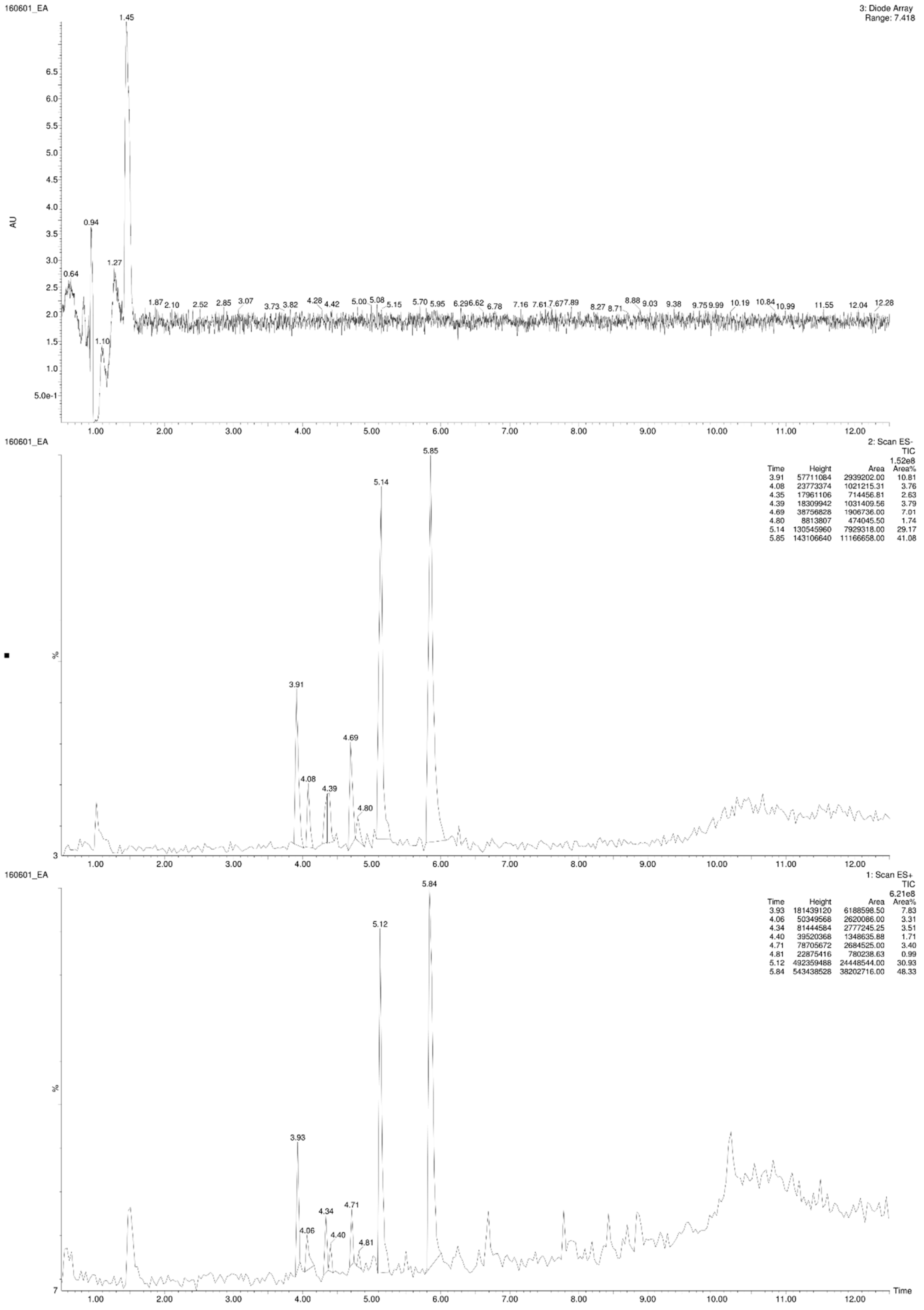

Figure S1. UPLC profile of the CIL1 saponin fraction.

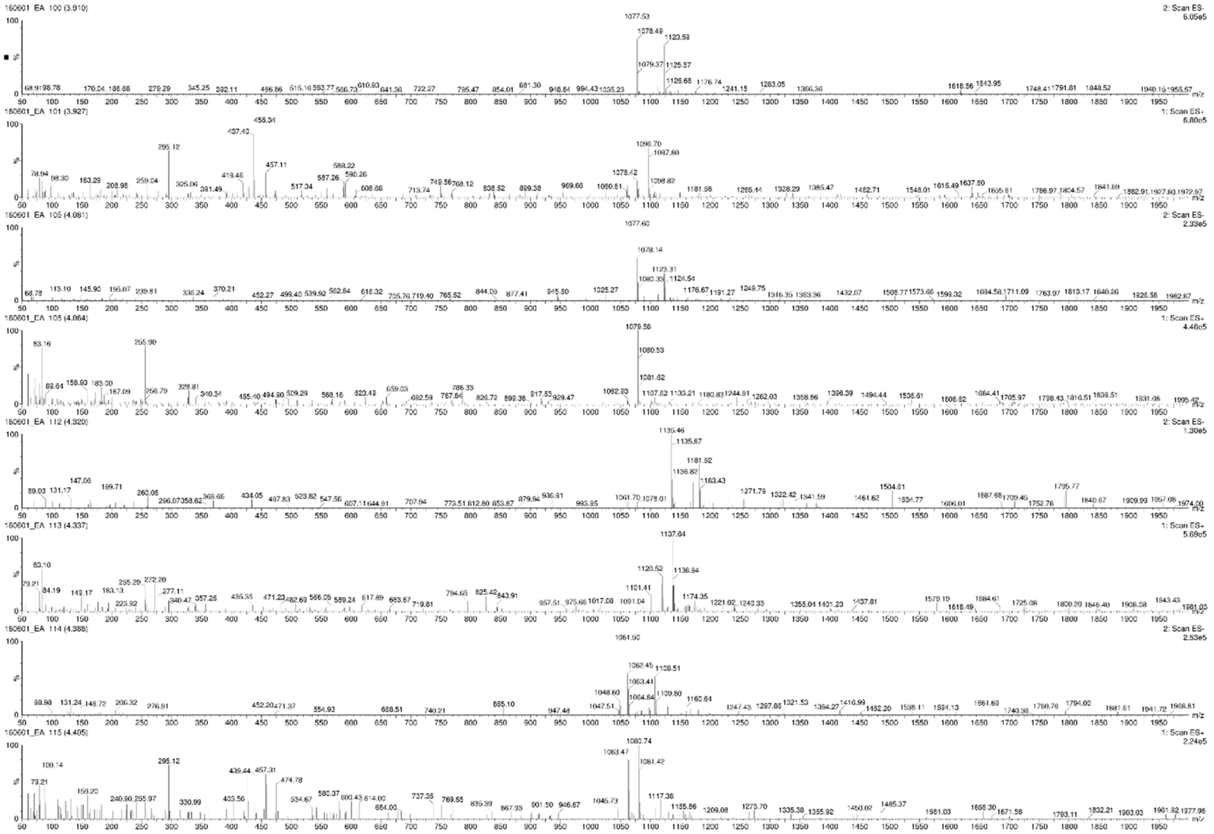

Figure S2. Mass profile of the CIL1 fraction components in negative ion mode.

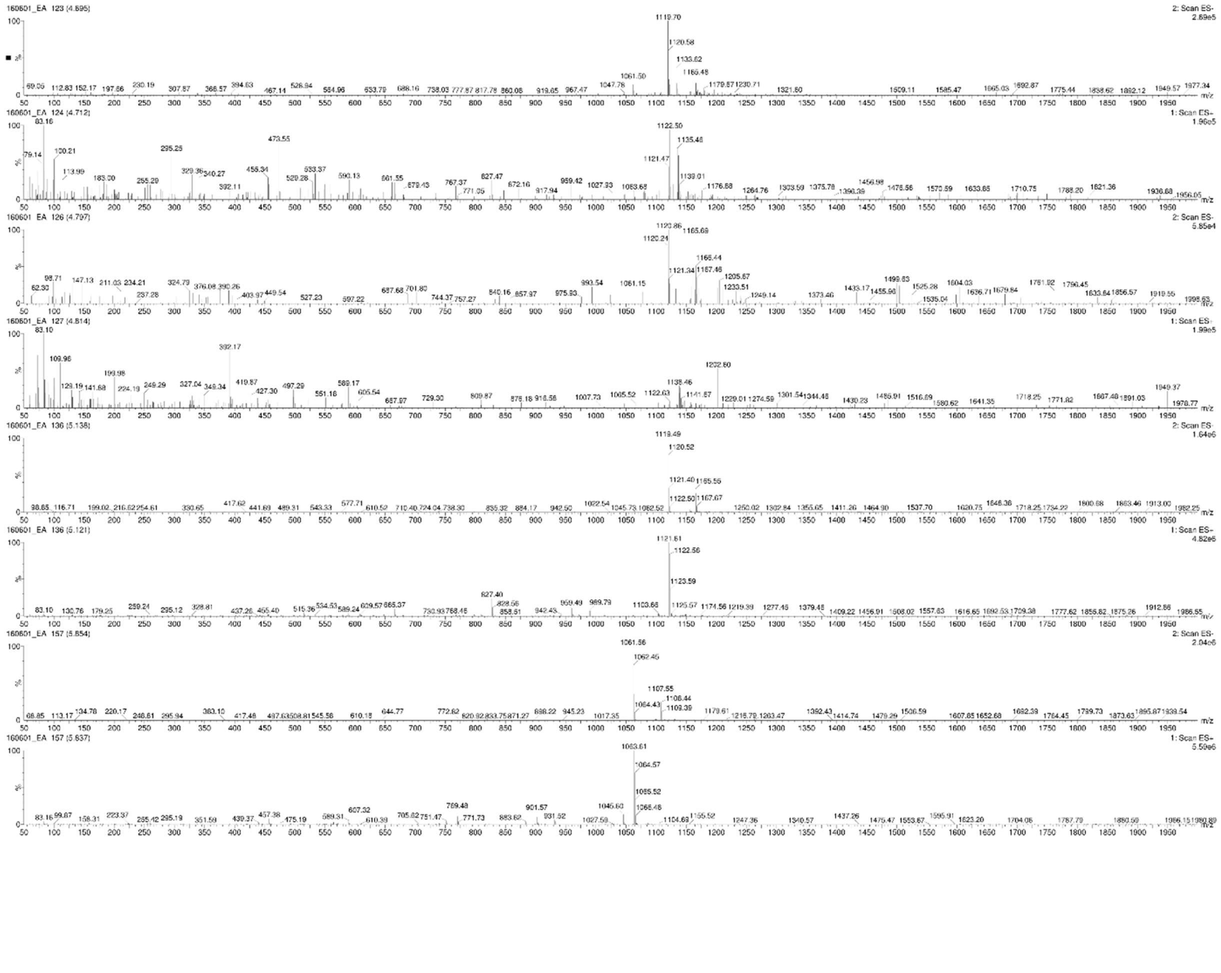

Figure S3. Mass profile of the CIL1 fraction components in negative ion mode (desglucoanagalloside B [M-H]- = 1061).

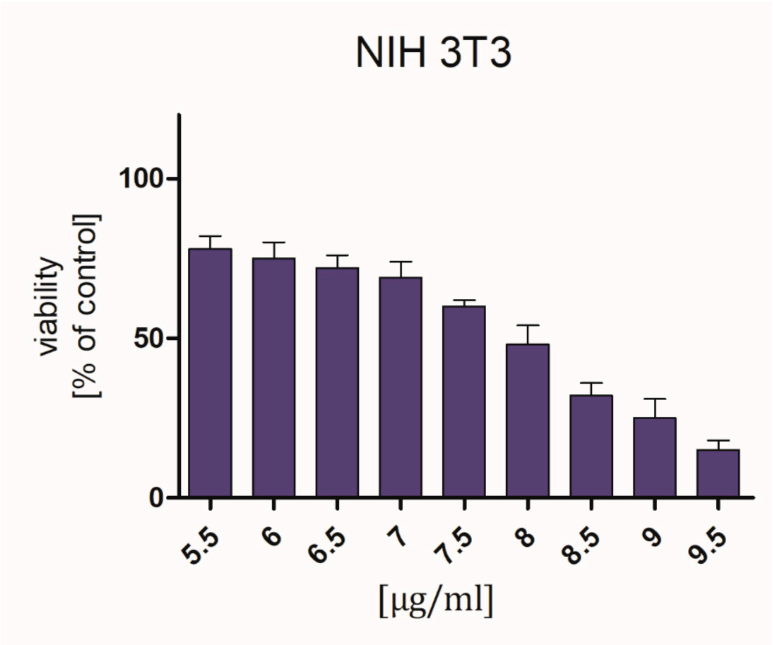

Figure S4. Viability of NIH3T3 cells after 72 h incubation in the presence of CIL1 in concentrations from 5.5 to 9.5 µg/ml. NIH 3T3 cells were seeded at an initial density of 2 × 10^3 cells / well in 96-well plates. Cells were incubated for 72 h with CIL1. The cell viability was determined using an MTT assay.

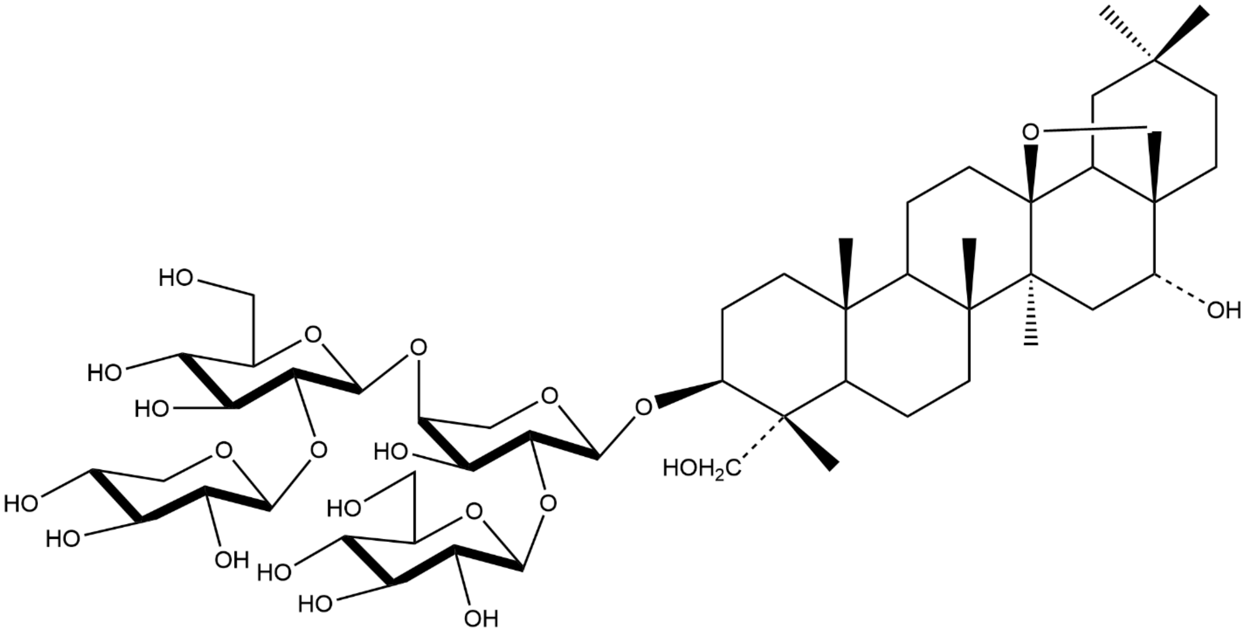

Figure S5. Chemical structure of the main component of the CIL1 fraction (desglucoanagalloside B).

Table S1. Influence of CIL 1 on the combined induction cytotoxicity by DE in HER14 and NIH3T3 cells in a CytoToxGlo assay. The table shows the IC_{50} values (nM) and the saponin-mediated enhancement factors (EF) for DE in HER14 and NIH3T3 cells. The receptor specificity brought about by CIL1 was therefore increased 355-fold.

	IC_{50} (nM)	EF
**DE_HER14**	0.79	
**DE_NIH3T3**	74.8	
**DE_HER14_CIL1**	0.000383	2062
**DE_NIH3T3_CIL1**	12.9	5.8
